# Advancing Bromegrass Breeding Through Imaging Phenotyping and Genomic Selection: A Review

**DOI:** 10.3389/fpls.2019.01673

**Published:** 2020-01-15

**Authors:** Dilip K. Biswas, Bruce Coulman, Bill Biligetu, Yong-Bi Fu

**Affiliations:** ^1^ Department of Plant Sciences, University of Saskatchewan, Saskatoon, SK, Canada; ^2^ Plant Gene Resources of Canada, Saskatoon Research and Development Centre, Agriculture and Agri-Food Canada, Saskatoon, SK, Canada

**Keywords:** abiotic stress, bromegrass species, crop adaptation, climate change, global warming, forage yield and quality, resource-use efficiency

## Abstract

Breeding forage crops for high yields of digestible biomass along with improved resource-use efficiency and wide adaptation is essential to meet future challenges in forage production imposed by growing demand, declining resources, and changing climate. Bromegrasses (*Bromus* spp.) are economically important forage species in the temperate regions of world, but genetic gain in forage yield of bromegrass is relatively low. In particular, limited breeding efforts have been made in improving abiotic stress tolerance and resource-use efficiency. We conducted a literature review on bromegrass breeding achievements and challenges, global climate change impacts on bromegrass species, and explored the feasibility of applying high-throughput imaging phenotyping techniques and genomic selection for further advances in forage yield and quality selection. Overall genetic gain in forage yield of bromegrass has been low, but genetic improvement in forage yield of smooth bromegrass (*Bromus inermis* Leyss) is somewhat higher than that of meadow bromegrass (*Bromus riparius* Rehm). This low genetic gain in bromegrass yield is due to a few factors such as its genetic complexity, lack of long-term breeding effort, and inadequate plant adaptation to changing climate. Studies examining the impacts of global climate change on bromegrass species show that global warming, heat stress, and drought have negative effects on forage yield. A number of useful physiological traits have been identified for genetic improvement to minimize yield loss. Available reports suggest that high-throughput imaging phenotyping techniques, including visual and infrared thermal imaging, imaging hyperspectral spectroscopy, and imaging chlorophyll fluorescence, are capable of capturing images of morphological, physiological, and biochemical traits related to plant growth, yield, and adaptation to abiotic stresses at different scales of organization. The more complex traits such as photosynthetic radiation-use efficiency, water-use efficiency, and nitrogen-use efficiency can be effectively assessed by utilizing combinations of imaging hyperspectral spectroscopy, infrared thermal imaging, and imaging chlorophyll fluorescence techniques in a breeding program. Genomic selection has been applied in the breeding of forage species and the applications show its potential in high ploidy, outcrossing species like bromegrass to improve the accuracy of parental selection and improve genetic gain. Together, these new technologies hold promise for improved genetic gain and wide adaptation in future bromegrass breeding.

## Introduction

Grazing land makes up approximately 60% of the world’s agricultural land, supporting 360 million cattle and more than 600 million sheep and goats (Food and Agriculture Organization (FAO), 2012). Forage production, commonly on marginal land that is less suited to grain production, contributes significantly to global food security (Mara, 2012). Although advances have been made in improving yield and quality of forage crops, further genetic improvement in forage yield and its stability under limited resources and changing environmental conditions remain as the important topics for future research. The impact of climate change on forage crops poses threats to future forage production and feed quality in temperate regions of the world (Tubiello et al., 2007; Chapman et al., 2012). Improvements in abiotic stress tolerance, resource-use efficiency, and yield can contribute to stable forage production to support sustainable animal production in the face of anticipated high population growth, resource limitation, and climate change.

Bromegrasses (*Bromus* spp.) are important cool season forage grasses in the temperate regions of the world used for hay, pasture, and land reclamation (Biligetu and Coulman, 2010). Breeding efforts have been focused on improving forage yield and quality of bromegrass species (Casler et al., 2000). However, genetic gains in forage yield over 90 years of conventional breeding efforts have been low due to the long breeding cycle, the difficulty of identifying superior parents from outcrossing populations (Casler et al., 2000; Vogel and Hendrickson, 2018), and the lack of efficient selection methods for increasing additive genetic variance between and within half-sib families (Casler and Brummer, 2008). On the other hand, little attention has been paid to improve resource-use efficiency and abiotic stress tolerance of this economically important forage species, although evidence from other kinds of crop indicates that crop adaptation to adverse environmental conditions is critical to thrive under changing climates (Challinor et al., 2014).

Genomic selection (GS) is a new breeding tool to enhance the rate of genetic gain by reducing the length of the breeding cycle and increasing selection accuracy (Meuwissen et al., 2001). GS is based on genome-wide molecular markers to predict genetic value of individual plants for a trait of interest, and has been effective in the prediction of the genetically complex traits (Fu et al., 2017) such as yield and abiotic stress tolerance. Recent advances in high-throughput imaging phenotyping tools along with low cost genetic technologies provide crop breeders with the potential to speed up the genetic gain in forage yield (Fahlgren et al., 2015). Imaging phenotyping techniques, including imaging fluorescence, thermal imaging, visible imaging, and hyperspectral imaging, are capable of screening a large number of genotypes for complex physiological traits related to growth, yield, and adaptation to biotic and abiotic stresses (Awada et al., 2018). Such phenotyping provides an extended opportunity to dissect the genetics of those quantitative traits in improving forage crops through GS.

An improved breeding efficiency through high-throughput phenotyping and GS can significantly increase genetic gains in abiotic stress tolerance, resource use efficiency, and forage yield of bromegrass and contribute to sustainable livestock production in the face of climate change. We therefore conducted a literature review on the progress of bromegrass breeding, and explored the feasibility of applying high-throughput imaging phenotyping techniques and GS for further advances in forage yield and quality of bromegrass under climate change. It is our hope that this review will update our knowledge of bromegrass breeding and research, and stimulate interest to apply new technologies to advance perennial grass breeding and genetic improvement.

## Bromegrass Breeding Achievements

**Figure 1:**
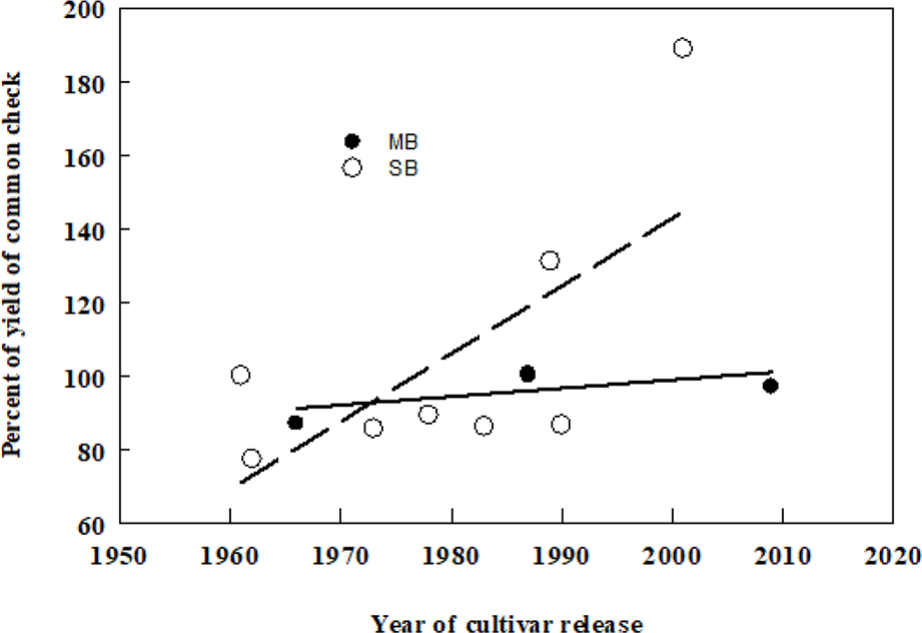
Associations between dry matter yields (DMY) of smooth (SB) and meadow (MB) bromegrass cultivars and their year of release in Canada. Yield of each cultivar has been averaged from multi-year yield trials conducted in the prairie provinces of Canada published in the Western Forage Test Reports (2000–2014) in western Canada. The common checks for SB and MB were Carlton and Fleet, respectively. DMY (SB) = −3,534 + 1.84 × year of release, d.f. = 7, r^2^ = 0.47, P = 0.059, DMY (MB) = −356 + 0.23 × year of release, d.f. = 3, r^2^ = 0.41, P = 0.360.

Smooth bromegrass (*Bromus inermis* Leyss) and meadow bromegrass (*Bromus riparius* Rehm.) are the two most widely cultivated species of the *Bromus* genus in North America. Smooth bromegrass (2n = 8x = 56), native to Eastern Europe and temperate Asia and meadow bromegrass (2n = 10x = 70), native to Southeastern Europe, the Caucasus, Turkey, and Central Asia were introduced to North America in 1884 and 1957, respectively. Smooth bromegrass is a leafy, deep rooted, and sod forming perennial, which is mainly grown for hay because of its higher sensitivity to defoliation (Biligetu and Coulman, 2010). Meadow bromegrass is a long-lived perennial with short rhizomes that is generally grown for pasture due to its rapid regrowth capacity (Vogel and Hendrickson, 2018). However, the initial breeding efforts in smooth bromegrass were largely on the investigation of existing natural variation among the introduced germplasm sources that had become naturalized land races in North America (Casler and Carlson, 1995). The first two meadow bromegrass cultivars in Canada were released by Dr. R.P. Knowles at Agriculture and Agri-Food Canada, Saskatoon, Saskatchewan in 1987. Moreover, a hybrid bromegrass (*B. inermis × B. riparius*) breeding program was initiated in the 1980s at Agriculture and Agri-Food Canada, Saskatoon, SK by Dr. Knowles and continued by Dr. B. Coulman to incorporate desirable characteristics from smooth and meadow bromegrass into an inter-specific hybrid and led to the release of three hybrid bromegrass cultivars, including one released in 2018. The hybrid brome cultivars can serve as dual-purpose bromegrass types, both as a hay crop in spring and as a pasture crop in the summer and fall (Knowles and Baron, 1990; Coulman and Knowles, 1995; Ferdnandez and Coulman, 2000; Coulman, 2004). Over all, genetic gain in forage yield over time of smooth brome has been somewhat higher than that of meadow bromegrass (**Figure 1**).

Grass breeders have developed nine smooth bromegrass cultivars, six meadow bromegrass, and three hybrid brome cultivars in Canada (**Table 1**). In the United States, grass breeders have developed 16 smooth bromegrass, five meadow bromegrass, and one hybrid bromegrass cultivars (**Table 2**). These bromegrass cultivars were generally selected for high pasture/hay yield, but some cultivars were selected for forage quality and specific adaptive traits. These released cultivars have greatly contributed to livestock production, along with soil conservation and improved grazing land productivity in the Northern Great Plains of the United States and Canada. However, Casler et al. (2000) conducted a comparative study with 30 smooth bromegrass cultivars or populations developed between 1942 and 1995 and revealed the slow breeding progress in smooth bromegrass in North America. The cultivars developed after 1942 had 540 kg/ha higher mean forage yield (i.e., 7%) than the cultivar “Lincoln,” which was selected for higher forage yield from introduced smooth bromegrass strains in the United States in 1942. This slow yield improvement could be partly explained by the fact that seven cultivars have been developed largely by selection within Lincoln, Saratoga, or Magna, but none of these cultivars had annual total forage yields significantly higher than their parent cultivars. Also, a number of breeding programs have emphasized selection for increased forage nutritive value rather than forage yield (Casler et al., 2000). Additional factors also include the difficulty in improving traits under complex polyploid inheritance and lower funding for public and private breeding programs than for other crop species (Casler et al., 2000; Vogel and Hendrickson, 2018).

**Table 1:** List of bromegrass cultivars with their characteristics and year of release in Canada.

Year of release	Cultivar	Characteristics	Developer
(a) Smooth bromegrass			
1936	Parkland		L.E. Kirk and T.M. Stevenson
1961	Carlton	High forage and seed yield	R.P. Knowles
1973	Magna		R.P. Knowles
1975	Tempo		W. Childers
1983	Signal		R.P. Knowles
1983	Bravo		
1990	Radisson	Good forage yield and quality	J. Surprenant and R.P. Knowles
2001	AC Rocket		R. Michaud
2014	AAC Royal		
(b) Meadow bromegrass			
1987	Fleet	High seed yield and quality	R.P. Knowles
1987	Paddock	Early maturity	R.P. Knowles
2009	Armada	High seed and forage yield	B. Coulman
2009	Admiral	High vigor and fall greenness	B. Coulman
2016	AAC Maximus	High forage yield	B. Coulman
(c) Hybrid bromegrass			
2000	AC Knowles	Dual-purpose, high yield, good regrowth, and fall greenness	B. Coulman
2003	AC Success	Dual-purpose, high yield, good regrowth, and early spring growth	B. Coulman
2018	AAC Torque	Dual-purpose and high forage yield	B. Coulman and B. Biligetu

**Table 2:** List of bromegrass cultivars with their characteristics and year of release in the United States.

Year of release	Cultivar	Characteristics	Developer
(a) Smooth bromegrass			
1942	Lincoln	High yield	L.C. Newell
1943	Manchar		
1950	Lyon		L.C. Newell
1950	Lancaster		L.C. Newell
1951	Homesteader		J.G. Ross
1955	Saratoga		
1962	Baylor		
1973	Barton		
1976	Beacon		
1978	Rebound	High regrowth yield and rapid recovery	J.G. Ross
1979	Cottonwood		
1979	Jubilee		
1989	York	High regrowth yield and rapid recovery	
1990	Badger		
1995	Alpha	High survival rate and persistence	
2014	Newell	High yield and forage digestibility	K.P. Vogel
(b) Meadow bromegrass			
1966	Regar		
2000	Montana		
2001	MacBeth		
2004	Cache	High yield, persistence and regrowth	K.V. Jensen
2015	Arsenal	High seedling emergence, yield, and forage quality	K.V. Jensen
(c) Hybrid bromegrass			
1965	Polar	Superior cold tolerance	

Regrowth capacity following cutting or grazing is an important forage trait for improved pasture production. It appears that there has been little or no improvement in regrowth forage yield in most of the smooth bromegrass cultivars released after 1942 except for three cultivars (i.e., Saratoga, Rebound, and York which were released in 1955, 1978, and 1989, respectively). It should be noted that York is selected from Rebound, which had been selected from Saratoga for regrowth forage yield. Regrowth forage yield of these three cultivars suggests that there has been progressive improvement in regrowth capacity of the smooth brome cultivars released between 1978 and 1989.

Forage quality has a direct effect on animal performance, forage value, and ultimately on profit of an operation. Breeding progress in forage quality of smooth bromegrass is often considered to be associated with a reduction of forage yield. However, Casler et al. (2000) evaluated breeding progress in bromegrass forage quality and showed that selections for increased *in vitro* dry matter digestibility have resulted in an average increase in *in vitro* dry matter digestibility of 9 g kg^−1^ (1.4%), an increase in forage yield of 330 kg ha^−1^ (5%), and a decrease (−1.2%) in neutral detergent fiber (NDF) of −8 g kg^−1^ in the cultivars released after 1942. The authors concluded that simultaneous improvement in yield and quality is possible and recurrent selection for improved forage quality traits such as *in vitro* dry matter digestibility, would have little, or no effect on forage yield of smooth bromegrass (Ehlke et al., 1986; Carpenter and Casler, 1990).

## Challenges of Bromegrass Breeding

Genetic gain in forage yields of bromegrass is low despite over nine decades of breeding efforts (Casler et al., 2000; Vogel and Hendrickson, 2018). Generally, breeding perennial forage crops for high yield is complicated with long breeding cycles and inability to exploit heterosis in commercial cultivars (Casler, 1998; Humphreys, 1999). Also, cultivars of forage species have been selected simultaneously for a wide array of economically important traits (i.e., improved quality, disease resistance, winter hardiness etc.), which are not specifically correlated, or may be negatively correlated, with forage yield (Casler, 2001). The low genetic gain in forage yield of perennial forage can be partly due to inefficient selection methods that make little use of additive genetic variance between and within half-sib families (Casler and Brummer, 2008). In addition, molecular and genomic research for improvement of stress tolerance in forage species is largely lacking (Zhang et al., 2006) for several reasons. The majority of perennial grass species including bromegrass are out-crossing and polyploidy. Forage yield and abiotic stress-resistance traits are regulated by a number of physiological processes and many genes (Yamada et al., 2004). Well-characterized genetic materials are not available. Repeatable and efficient phenotyping protocols for many targeted traits have not been developed.

Abiotic stress is the primary cause of crop loss worldwide reducing yield of major crops by 50% (Boyer, 1982). Breeding of crops to minimize yield loss is an important step for stable forage production in the face of climate change. However, traditional approaches to breeding crop plants with improved abiotic stress tolerances have so far met limited success (Richards, 1996) for several factors. They include i) the focus on yield rather than on specific tolerance traits, ii) the difficulty of choosing appropriate selection environments in highly variable target environments, iii) the difficulty in breeding of tolerance traits, which are strongly affected by genotype × environment interactions, iv) the limited efficiency in plant phenotyping, v) relatively infrequent use of physiological traits as measures of tolerance, vi) the complex genetics of abiotic stress tolerance traits, and vii) the limitation in acquisition of desired traits from closely related species (Richards, 1996; Tester and Bacic, 2005; Reynolds and Trethowan, 2007; Ghanem, 2015).

## Assessing Global Climate Change Impacts on Bromograss

### Effects of Warmer Temperature on Forage Yield and Quality

Temperature regulates the local adaptation of perennial forage crops by governing important physiological processes such as vernalization, flowering, and cold acclimation and determines winter survival and seasonal yield distribution. It has been reported that the risks of winter injury/frost damage to perennial forage crops in eastern Canada would likely increase due to less cold hardening during fall and reduced snow cover during the cold period under predicted warmer than normal fall and winter temperatures (Belanger et al., 2002). Reyes-Fox et al. (2016) reported that warming advanced leaf emergence and flower production, but expedited seed maturation and leaf senescence at the species level of a mixed-grass prairie plant community. Whittington et al. (2015) conducted a 3-year field warming study in a temperate grassland to investigate the effects of two levels of warming (+ ~1.5 and + ~3°C) on the phenology of budding, flowering onset, and peak flowering of 10 perennial plant species at both individual and population scales. They found that warming led to high normalized vegetation index values in the spring, indicating that warming accelerated spring biomass growth, but did not significantly affect senescence of grass species. Thus, warming has variable responses on leaf senescence of perennial forage species and an accelerated leaf senescence would have negative effects on forage yield, seasonal yield distribution, and forage quality. Temperature is also the most influential factor on the nutritive value of forages as it alters developmental stage of plants and the time of harvest (Buxton and Fales, 1994). Wilson et al. (1991) demonstrated that warming altered structural carbohydrates and tissue digestibility differently in leaves and stems. Similarly, Bloor et al. (2010) reported that warming reduced digestibility of forage as warming accelerated plant growth with an increase in NDF, acid detergent fiber, and lignin concentrations while it reduced leaf: stem ratio. Bai et al. (2013) conducted a meta-analysis on the effect of experimental warming on terrestrial ecosystems and reported that warming slightly increased plant nitrogen (an indicator of crude protein). In contrast, another meta-analysis on the effect of climate change on forage quality showed that warming had no effect on nitrogen, water soluble carbohydrates, structural carbohydrates, and digestibility (Dumont et al., 2015).

### Effects of High Temperature Stress on Forage Yield and Quality

Temperatures above the normal optimum result in high temperature/heat stress, which negatively impacts crop growth and development by disrupting regular plant functions, including molecular, physiological, and anatomical processes, ultimately reducing crop production (Reynolds and Trethowan, 2007; Mathur et al., 2014; Biswas et al., 2019). Sprague (1943) reported reduced germination of several perennial grass species, including bromegrass at temperatures above 29°C. Sloan (1941) reported significant differences in the response of a number of grasses, including bromegrass to heat stress and found a positive correlation between the percentage of tissue injured by the exposure to high temperature and the number of dead seedlings caused by the heat treatment. The author also added that bromegrass strains of Kansas origin exhibited the highest degree of tolerance to heat stress. Patterson (1940) reported highly significant differences in high temperature tolerance in progeny groups growing in a nursery, but there was no relationship between the agronomic characters of vigor and forage production and resistance to high temperatures. The impacts of heat stress on three brome species (mountain brome, *Bromus marginatus*; prairie brome, *Bromus catharticus*; Harlan brome, *Bromus stamineus*) were examined between 7 and 70 days. The results indicate that bromegrasses were sensitive to heat stress until approximately 28 days, then heat tolerance gradually increased with plant age. Heat stress causes reductions in photosynthetic rate, chlorophyll content, cell membrane stability, and carbohydrate accumulation in a number of forage grass species (Jiang and Huang, 2000). Nutritive value of forage grasses is reduced at high temperatures likely driven by a combination of changes to species identity and changes to physiology and phenology (Lee et al., 2017). Extreme climatic events such as heat stress can lead to tissue senescence that can strongly decrease forage quality. Moderate heat stress results in faster plant maturation, decreased water content of plant tissues, and increased water soluble carbohydrates. Heat stress-induced rapid maturation of plants also reduces leaf-to-stem ratio and increases cell wall content, including lignin which interferes with the digestion of cell wall polysaccharides by acting as a physical barrier to microbial enzymes (Moore and Jung, 2001). Consequently, heat stress usually decreases dry matter digestibility (Lu, 1989). Heat stress is also found to both increase and decrease crude protein in forage grasses (Dumont et al., 2015; Lee et al., 2017).

### Effects of Drought on Forage Yield and Quality

Drought accelerates leaf senescence and reduces growth and forage yield of cool-season perennial forage grasses. Forage grass species vary widely in sensitivity of leaf senescence to drought (Bittman et al., 1988). Leaf senescence is generally thought to contribute to drought avoidance by reducing transpiration and active leaf area. Despite having much greater leaf area, vegetative tillers of smooth bromegrass underwent less leaf senescence during drought than reproductive tillers (Bittman et al., 1988). This suggests that drought advances the maturity of reproductive tillers, but not vegetative tillers in bromegrass. The range of water potential that induces leaf senescence in bromegrass is −1.85 to −2.25 MPa (Bittman et al., 1988). The seasonal productivity of grass species is influenced by rate and extent of recovery from soil water deficit under intermittent drought. Bittman and Simpson (1987) studied drought tolerance and drought recovery capacity of several perennial grass species including smooth bromegrass and found that smooth bromegrass species maintained green leaf tissue during drought, allowing assimilation and growth to continue rapidly after re-watering. Smooth bromegrass developed its leaf area rapidly, although somewhat later than crested wheatgrass (*Agropyron cristatum* L.). Bittman and Simpson (1989) compared stomatal conductance of bromegrass and other perennial grass species under field drought and non-drought conditions, and reported that bromegrass species had lower stomatal conductance than other species under both drought and non-drought conditions. This suggests that bromegrass species might have higher transpiration efficiency that could contribute to maintaining high leaf water potential and growth under drought conditions. Bahrani et al. (2010) examined the effects of two levels of soil water stress on 10 forage grass species, both native and introduced to Iran, and found that increased water stress decreased plant height, leaf water potential, leaf area, root dry weight, water use efficiency, and total dry matter production in all species with varying degree of reductions among species. However, the authors concluded that smooth bromegrass and tall wheatgrass [*Thinopyrum ponticum* (Podp.) Z. –W. Liu & R. –C. Wang] were the most drought tolerant species in terms of total dry matter production. Sheaffer et al. (1992) conducted a controlled drought study to explore the effect of drought on regrowth capacity of four cool season perennial grasses including smooth bromegrass. The authors reported that smooth bromegrass had the lowest forage yield when drought occurred throughout the regrowth cycle, but showed the highest seasonal forage yield among the four species. This implies that regrowth of smooth brome was most sensitive to drought, although it had the highest compensatory growth following drought stress. Saeidnia et al. (2017a) conducted a field drought study with 36 genotypes of clonally propagated smooth bromegrass and found that water stress had negative effects on seed yield and its components, but reduced genotypic variation of measured traits. The authors found that, on average, water stress reduced seed and forage yield by 38 and 14%, respectively, suggesting a higher impact of drought on reproductive growth than vegetative growth of smooth bromegrass genotypes. In a 2-year field drought study with 36 smooth bromegrass genotypes selected from 25 half-sib polycross progenies, drought overall reduced dry matter yield of smooth brome genotypes by 36 and 39%, relative to non-drought control in 2013 and 2014 (Saeidnia et al., 2017b). Reduction of dry matter yield in bromegrass under drought stress can be explained by reduction in photosynthetic capacity as evidenced by drought-caused reduction in photosynthetic pigments and relative water content.

Drought affects forage quality by altering growth and physiological processes. Bittman et al. (1988) reported that while drought increased leaf senescence, it slightly improved digestibility by lowering acid detergent fiber and acid detergent lignin in smooth bromegrass. Sheaffer et al. (1992) examined drought effects on several species of cool season perennial forage grasses, including smooth bromegrass, and reported that drought enhanced forage quality of smooth bromegrass as documented by increased crude protein concentration in leaf, stem, and total forage and decreased NDF and acid detergent fiber concentrations when drought occurred throughout growth period. In contrast, water deficit may increase the rate of seasonal decline in nitrogen and phosphorus concentrations in forage, probably due to reduced uptake of nitrogen and phosphorus by reducing transpiration and increasing leaf senescence as found in the drought-stressed rapeseed (*Brassica napus* L.) plants (Biswas et al., 2019). However, drought improves the digestibility of forages by reducing the rate of increase in acid detergent fiber and lignin. Since fiber and lignin are major components of the cell wall, the reduced rate of increase in these two cellular components under drought might be due to drought-induced reduction in cell number and cell expansion. This result gains further support from a meta-analysis on the effects of drought on forage quality in which the authors reported that drought led to an average 5% decrease in plant cell-wall (i.e., NDF) content. Therefore, digestibility of forage under drought increased on average by 10%, with strong variation between experiments (Skinner et al., 2004; Craine et al., 2010). Results from line source sprinkler designs indicate that, as water stress increased, the concentrations of crude protein, NDF digestibility, and *in vitro* true digestibility values increased, but water stress had no effect on NDF value of forage (Asay et al., 2002; Jensen et al., 2008).

## Phenotyping Bromegrass Using High-Throughput Imaging

### High-Throughput Phenotyping With Imaging Techniques

**Figure 2:**
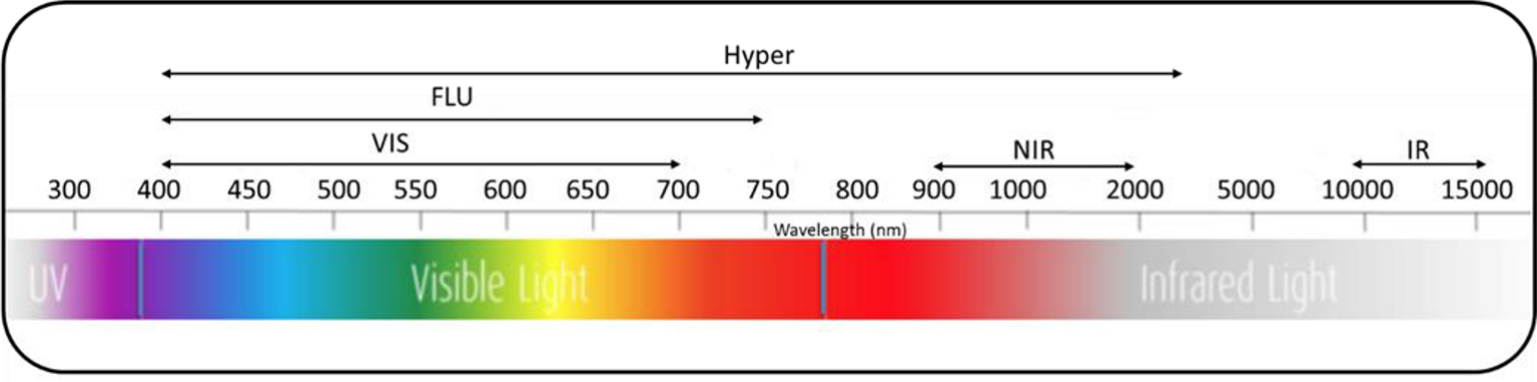
Spectra of different imaging sensors used for high-throughput imaging plant phenotyping. VIS, visual; FLU, fluorescence; Hyper, hyperspectral; NIR, near-infrared; IR, infrared.

High-throughput imaging technologies utilize different sensors and spectra (**Figure 2**) to image morphological, physiological, biochemical, and growth characteristics of plants. For instance, visible imaging is used to estimate morphological traits, crop phenology, and shoot biomass. Imaging chlorophyll fluorescence is used to evaluate photosynthetic efficiency, photoprotection, and oxidative stress. Hyperspectral imaging is used to identify biochemical physiological changes induced by environmental and nutrient stresses. Thermal imaging is used to detect water and heat stresses (Awada et al., 2018). High-throughput imaging phenotyping techniques are particularly useful to dissect complex traits and physiological mechanisms by capturing and analyzing spatial and temporal variability of plant responses (Fiorani and Schurr, 2013). Different imaging sensors including visible, fluorescence, thermal, and hyperspectral imaging, along with open sourced image processing and trait extraction tools, are capable of screening germplasm collections for desirable traits in a breeding program (Araus and Cairns, 2014; Fahlgren et al., 2015; Awada et al., 2018; Araus et al., 2018). As a result, high-throughput imaging techniques are now empowering various research approaches such as genome-wide association studies to minimize the genotype-phenotype gap by identifying associations between phenotypic traits and DNA markers across a range of genotypes (Slovak et al., 2014; Zhang et al., 2017).

### Phenotyping for Photosynthetic Efficiency and Forage Yield

Forage yield increase of bromegrass needs to exceed the current rate of genetic gains to meet the projected demand for high forage yields by livestock industries in the future. Forage yield is mainly determined by two processes: i) the interception of incident solar irradiance by the crop canopy, which depends on the photosynthetic area of the canopy; and ii) the conversion of the intercepted radiant energy to potential chemical energy, which relies on the overall photosynthetic efficiency of the crop (Hay and Walker, 1989). Photosynthetic efficiency by which a crop captures light energy utilizing CO_2_ and water, and converts it to biomass over the growing season is a key determinant of crop yield (biomass/grain) (Long et al., 2006). Further improvements in forage yield of bromegrass require an increase in biomass through improvements in photosynthetic traits and photosynthetic radiation-use efficiency. Photosynthesis-related traits such as nitrogen per unit leaf area and leaf dry mass per area are normally measured with laborious, destructive, laboratory-based methods, while physiological and biochemical traits underpinning photosynthetic efficiency, such as maximum rubisco activity and electron transport rate are measured using time-consuming laboratory analysis and/or gas exchange measuring tools. Thus, the high cost and considerable time required by testing on a breeding scale explains partly the limited use of selection for physiological and biochemical characteristics of crop species in a breeding program.

As an alternative, canopy reflectance obtained from hyperspectral cameras is associated with specific plant characteristics and has been proposed as a fast and non-destructive technique that can be efficiently used in breeding programs (Babar et al., 2006; Gizaw et al., 2018). For instance, infrared spectral reflectance of leaves has been correlated with maximum rubisco activity and electron transport rate, and have been used to predict photosynthetic efficiency of tree species grown in a glasshouse (Serbin et al., 2012), and field-grown soybean [*Glycine max* (L.) Merr.] (Ainsworth et al., 2014). Similarly, Silva-Perez et al. (2018) have constructed predictive models using gas exchange and hyperspectral reflectance (350–2,500 nm) of leaves from 76 wheat (*Ttriticum aestivum* L.) genotypes grown in glasshouses and reported correlation coefficients (r^2^) of maximum rubisco activity, electron transport rate, leaf dry mass per area, and nitrogen per leaf area are 0.62, 0.7, 0.81, 0.89, and 0.93, respectively. Moreover, photochemical reflectance index, which provides a linkage with photosystem II efficiency by tracking the variation in xanthophyll cycle pigments, can be used successfully to assess photosynthetic function and radiation-use efficiency. In fact, high-throughput assessment of radiation-use efficiency by canopy reflectance can be used to assess genetic variation in photosynthetic efficiency mechanisms on a breeding scale (Serbin et al., 2012; Yendrek et al., 2017). The results suggest that high-throughput hyperspectral imaging might facilitate identification of molecular markers and candidate genes underpinning genetic variation in photosynthetic efficiency and photosynthesis related traits of bromegrass species.

Conventional phenotyping (visual scoring of plants and destructive samplings for biomass at different growth stages) of forage dry matter yields of a large number of breeding materials is laborious and time-intensive. An intensive selection based on high-throughput imaging techniques could accelerate genetic gains in forage dry matter yield. Available reports indicate that assessments of grain yield with near infrared canopy reflectance using field-portable spectroradiometers have been useful for direct selection of small-grain cereal crops in the field (Reynolds et al., 2009; Araus and Cairns, 2014). A number of indices, including normalized difference in vegetation index and reflectance ratios, can be derived from the canopy reflectance data obtained from hyperspectral cameras to assess ground cover, growth, and biomass yield. For instance, biomass yields of Bermuda grass [*Cynodon dactylon* (L.) Pers] have been linearly correlated with the reflectance ratio of R915/R975 (0.44 < r^2^ > 0.63) as well as with the first derivatives of canopy reflectance with wavebands centered at 935 nm (0.49 < r^2^ > 0.68). Hence, the two-narrow-waveband reflectance ratios or the first derivatives in visible and near-infrared spectral regions are useful for real-time and non-destructive prediction of forage productivity in Bermuda grass pastures (Starks et al., 2006). These indicate that hyperspectral imaging techniques may be an alternative to the laborious destructive methods for estimating forage dry matter yield of bromegrass in small-plot field experiments. On the other hand, visual imaging-based indirect selection for morphological traits such as leaf area, plant height, tiller density, or plant vigour is also found to be correlated with forage dry matter yield of grass species (Majidi et al., 2009). The visual images of individual plants can be captured and they can be analyzed with image-processing tools to identify plant-derived pixels for measuring morphological (shape, structure), geometric (length, area), and color properties of each plant. Plant pixel area from a single image stack can be used to estimate total leaf area or plant volume that can accurately model fresh or dry weight of above ground biomass of crop plants. In addition, visual and hyperspectral imaging of plants at multiple time points over a growth period can be used to measure relative growth rate of plants and dynamic growth processes. These imply that both visual and hyperspectral imaging can be explored as high-throughput tools to assess photosynthetic efficiency, growth, and forage dry matter yield of bromegrass accessions.

### Phenotyping for Forage Quality

Forage quality is an important factor that affects animal health and performance. Generally, forage quality traits are determined by dry/wet procedures in the laboratory, which limits our capacity to screen a large number of accessions as well as to explore genetic variability of quality traits in a breeding program. Although near-infrared reflectance technology allows us to analyze quality traits in a relatively short period of time (Gillon et al., 1999), sample collection from the field and processing in the laboratory are time and labor-intensive procedure. Canopy reflectance has been demonstrated to be successful in determining forage quality traits in a number of studies. For example, Starks et al. (2004) have analyzed the relationships between canopy reflectance and the forage quality variables such as NDF, acid detergent fiber, and nitrogen concentration in Bermuda grass pastures using modified partial least square regression methods and reported a linear correlation between laboratory-based forage quality traits and pasture canopy reflectance indices. In another study, the concentration of crude protein and availability of crude protein in Bermuda grass have been successfully predicted by either the ratios or derivatives of canopy reflectance (Starks et al., 2006). Canopy reflectance of pastures measured with airborne hyperspectral imaging has also been found to be correlated with crude protein % (r^2^ = 0.80), organic matter digestibility (r^2^ = 0.85), and metabolic energy (r^2^ = 0.79) (Yule et al., 2015; Reddy et al., 2016). The available results on the associations between laboratory-based forage quality traits and canopy reflectance indices of different forage crops suggest that high-throughput hyperspectral imaging is practically feasible for evaluating forage quality traits of bromegrass.

### Phenotyping for Abiotic Stress Tolerance and Resource Use-Efficiency

It is difficult to breed crops with improved abiotic stress tolerance using traditional approaches as discussed above. Clearly, improved phenotyping efficiency and dedicated breeding programs using advanced phenotyping tools are required to improve crop abiotic stress tolerance to enhance stability of forage yield. Given the current lack of genetic markers for accurate evaluation of stress adaptation, physiological traits can be viewed as proxy genetic markers (Reynolds and Trethowan, 2007). These proxies are more feasible to apply molecular marker-based selection for targeted breeding objectives if the physiological and genetic basis of stress adaptation are identified. As a result, a core set of traits are proposed to improve bromegrass adaptation to global warming, heat, and drought stresses (**Tables 3** and **4**), which were summarized from a number of reports (Reynolds and Trethowan, 2007; Reynolds et al., 2009; Reynolds et al., 2015). Modulation of plant phenological development and leaf senescence can be beneficial to increase stability of forage yield under warmer environments. For example, a delay in flowering time will increase forage productivity and quality as global warming will advance leaf emergence, green up and flowering, but also accelerate leaf senescence. Available reports demonstrate that genetic modification of flowering time of forage grass species such as red fescue (*Festuca rubra* L.) and perennial ryegrass (*Lolium perenne* L.) is possible (Jensen et al., 2004; Wang and Forster, 2017). Similarly, delaying leaf senescence can further enhance seasonal forage productivity as stay-green phenotypes in wheat have shown improved wheat productivity under abiotic stresses such as drought and heat stresses (Vijayalakshmi et al., 2010). Thus, controlling flowering time and stay-green characteristics of bromegrass species may be important for improving yield and forage quality in changing environments. Also, visual imaging has been successfully employed for determining flowering time in field-grown cotton (*Gossypium hirsutum* L.) (Xu et al., 2019). A number of indices derived from canopy/leaf reflectance of infrared and near-infrared wavelengths from hyperspectral imaging data have been shown to be effective in measuring relative greenness, foliage development, chlorophyll content, and leaf senescence (Penuelas and Filella, 1998). Thus, a combination of visual and hyperspectral imaging can be explored for assessing germplasm for genetic variation in flowering time and stay-green phenotypes in bromegrass breeding programs.

**Table 3:** List of desirable traits with the potential to improve bromegrass adaptation to changing environment and heat stress.

Desirable trait	Effects	Level of adaptation
(a) Changing environment		
Plant phenological development	Delay in flowering to maintain active vegetative growth	Plant/crop
Delayed leaf senescence	Increase growing season and productivity by maintain active leaf area	Molecular
(b) Heat stress		
Deeper root	Extract soil water from deeper root zone	Organ
Cooler canopy	Maintain photosynthetic activity and productivity	Organ
Higher stomatal conductance	Enhance transpiration cooling	Organ
Higher carotenoids	Increase photoprotection of photosystem 2 by quenching of chlorophyll triplet states and scavenging of both superoxide and hydroxyl radicals	Molecular
Higher antioxidants	Increase detoxification of reactive oxygen species	Molecular
Rubisco activase	Protect thylakoid associated protein synthesis machinery against heat inactivation	Molecular
Higher radiation-use efficiency	Increase conversion of light energy and CO_2_ into biomass	Plant/crop/molecular

The physiological traits and their effects on crop plants are summarized from the reports published elsewhere (Reynolds and Trethowan, 2007; Reynolds et al., 2009; Reynolds et al., 2015).

**Table 4:** List of desirable traits for improving bromegrass adaptation to drought stress.

Desirable trait	Effects	Level of adaptation
Phenological development	Match drought conditions to crop stages that are relatively drought tolerant	Plant/crop
Deeper root	Extract soil moisture from deeper root zone	Organ
Higher stomatal regulation	Reduce water loss through transpiration	Organ
Leaf rolling	Reduce radiation load and transpiration	Organ
Higher wax deposition	Reduce radiation load by reflecting and non-stomatal water loss	Molecular
Delayed leaf senescence	Maintain photosynthetic activity and productivity	Molecular
Higher osmotic adjustment	Maintain leaf turgor and membrane stability	Molecular
Higher carotenoids	Increase thermal dissipation	Molecular
Higher antioxidant capacity	Increase detoxification of reactive oxygen species	Molecular
Higher rubisco specificity	Increase carboxylation capacity under low CO_2_ concentration when stomata are partially closed	Molecular
Higher water-use efficiency	Maintain photosynthetic productivity with minimum soil water used	Plant/crop/molecular

The physiological traits and their effects on crop plants are summarized from the reports published elsewhere (Reynolds and Trethowan, 2007; Reynolds et al., 2009; Reynolds et al., 2015).

Breeding crops for improved adaptation specifically to heat and drought can help to increase/sustain genetic gains for crop yields under climate change (Reynolds et al., 2009). In general, plant adaptation to abiotic stresses such as heat and drought stress requires substantial improvement in the capacity of plant to access soil water, photo-protection of photosystem II, and water/radiation-use efficiency (Richards, 1996). Deeper root systems and higher root growth that permit better access to soil water have obvious benefit under drought, while enabling heat-stressed canopies to match the high evaporative demand associated with hot and low-relative humidity environments, resulting in higher transpiration rate and a cooler canopy (Reynolds and Trethowan, 2007). Selection for highly regulated stomatal function can improve water-use efficiency under drought conditions as effective stomatal regulation allows leaves to avoid low water potentials (Brodribb et al., 2009). High regulation of stomatal functions and cooler canopy under drought and/or heat stress can be measured by infrared thermal imaging, which is now an established technology for high-throughput phenotyping of plants for differences in stomatal behavior (Jones et al., 2009; Reynolds et al., 2015). For example, infrared thermal imaging techniques are being used routinely to identify cooler crop canopies in wheat breeding program under rainfed environments to enrich alleles associated with improved adaptation to drought (Reynolds et al., 2015). In addition, a number of spectral indices have been proposed to estimate water content of leaf tissues remotely as a measure of drought stress (Chen et al., 2005; Seelig et al., 2008). High-throughput hyperspectral imaging can also be used to screen germplasm in the field for genetic variation in cellular osmotic adjustments that contribute to drought tolerance in plants.

Photoprotection of photosystem II is mediated by a number of photoprotective mechanisms such as: i) carotenoids that dissipate excess energy as heat or quench reactive oxygen species; ii) antioxidant systems that detoxify reactive oxygen species; and iii) wax deposition on leaves that reduces radiation load by reflecting a part of intercepted radiation. These mechanisms are likely to be important under drought and/or heat stress as insufficient water or impaired metabolism impedes full utilization of light energy leading to higher oxidative damage to photosynthetic machinery (Mittler and Zilinskas, 1994; Niyogi, 1999). Photoprotective carotenoids and wax are therefore correlated with drought tolerance of crop plants, including bromegrass (Saeidnia et al., 2017b), and these traits can be measured using canopy/leaf reflectance data derived from high-throughput hyperspectral imaging (Penuelas and Filella, 1998). Higher photoprotective capacity generally contributes to increased quantum efficiency of photosystem II, which can be measured using high-throughput imaging chlorophyll fluorescence. The level of non-photochemical quenching coefficient is considered as photoprotective capacity in plants exposed to abiotic stresses and can be measured using imaging chlorophyll fluorescence (Harbinson et al., 2012). Selection for high quantum efficiency essentially can improve photosynthetic radiation-use efficiency for CO_2_ fixation and dry-matter accumulation under drought stress. Radiation-use efficiency and photosynthetic water-use efficiency are generally complex physiological traits and their improvement requires improvement of multiple mechanisms at different scales of organization (**Table 4**). An improvement in carboxylation capacity, photoinhibition, and optimizing stomatal regulation and canopy structure can substantially increase radiation-use efficiency/photosynthetic water-use efficiency and crop yields under conditions of heat and/or drought stress (Simkin et al., 2019). The biochemical, physiological, and morphological traits related to resource-use efficiency of bromegrass species can be improved through utilization of a combination of hyperspectral imaging, imaging chlorophyll fluorescence, infrared thermal and visual imaging techniques in a breeding program. However, a little attention was paid to explore the potential benefits of these imaging techniques to forage breeding.

**Figure 3:**
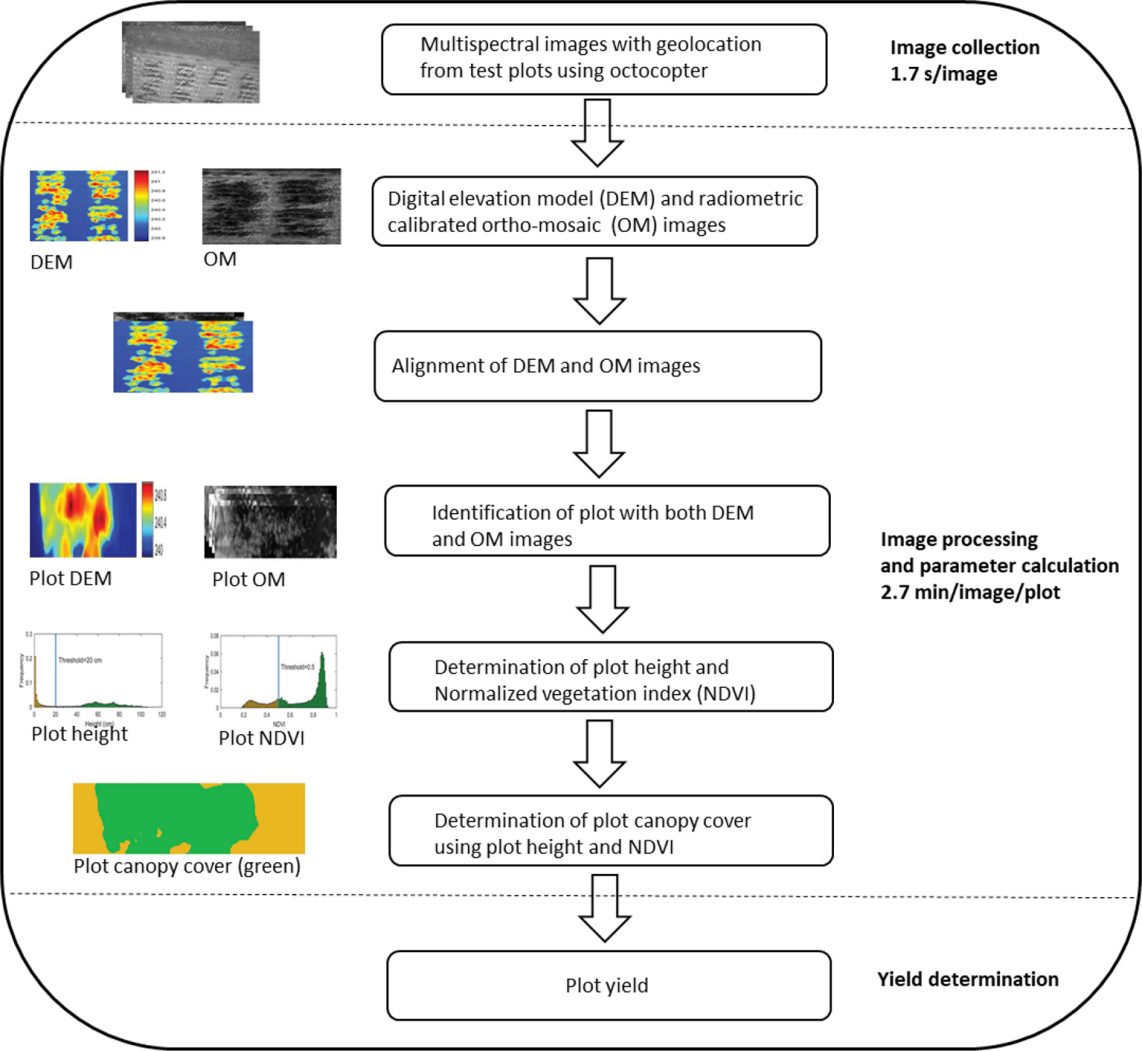
An illustration of major steps in the application of multispectral camera to phenotype cotton canopy cover/yield in the field (adopted from Xu et al., 2019).

High-throughput imaging phenotyping techniques are increasingly being used in crop improvement (Araus and Cairns, 2014; Xu et al., 2019; Loladze et al., 2019; Juliana et al., 2019). The most successful study was the application of multispectral camera by Xu et al. (2019) to phenotype cotton accessions for canopy cover, as illustrated in **Figure 3**. The application was not only efficient in terms of reduced time, but also a strong correlation (R^2^ = > 0.92) was found between calculated and measured maximum plant heights and a moderate association (R^2^ = 0.32–0.57) between normalized vegetation index and canopy cover. Similarly, Loladze et al. (2019) also applied hyperspectral and infrared thermal cameras to phenotype grain yield in maize (*Zea mays* L.) with different levels of disease infection. The study not only showed strong relationships between grain yield, vegetation index, and canopy temperature under disease pressure (**Table 5**), but also demonstrated that imaging techniques could help reduce the time and cost required for the development of improved maize germplasm. These studies, together, suggest that the high-throughput imaging techniques can be successfully applied to phenotype a large number of crop germplasm, including bromegrass breeding materials, for improvement of forage yield, quality, and abiotic stress tolerance.

**Table 5:** Published applications of high-throughput imaging phenotyping techniques to crop genetic improvement.

Traits	Crop	Study objectives	Number of accession	Imaging sensor	Imaging environment	Reference
Plant height, canopy cover, vegetation index, flowering time	Cotton	Phenotypic analysis of breeding materials	240	Visual, near-infrared (octocopter)	F	Xu et al. (2019)
Biomass, green leaf area, chlorophyll content	Rice	GWAS study for natural variation	529	Hyperspectral	CE	Feng et al. (2017)
Growth, chlorophyll content/senescence	Rice	Dissection of genetic architecture of temporal salinity response	373	Visual, fluorescence	CE	Campbell et al. (2015)
Green leaf area	Bread wheat	GWAS study for grain yield	4368	Hyperspectral (Clipper aircraft)	F	Juliana et al. (2019)
Grain yield, vegetation index, canopy temperature	Maize	Phenotyping for foliar diseases tolerance	25	Visual, infrared	F	Loladze et al. (2019)

F, field; CE, controlled environment.

## Genetic Improvement of Bromegrass Using Genomic Selection

### Genomic Selection

GS was developed in 2001 by taking advantage of genome-wide random genetic markers to predict the genomic estimated breeding value (GEBV) of individuals for a given trait of interest (Meuwissen et al., 2001). The trait predication assumes that all genes (with either large or small effects) affecting a targeted trait are in linkage disequilibrium with some assayed markers that are distributed across the genome, offering better genetic estimation of a trait value for individuals (Meuwissen, 2007). Selecting new breeding parents can be made based on individual GEBV estimates. This selection procedure could lead to a shorter breeding cycle duration as there is no requirement for phenotyping of quantitative traits of late filial generations in inbred annuals, and in the perennials, a multi-year evaluation for each generation may not be needed (Kumar et al., 2011; Bassi et al., 2016). With the advances in high-throughput phenotyping technologies and flexible, low-cost single nucleotide polymorphism marker platforms, such as genotyping-by-sequencing (GBS) (Elshire et al., 2011), GS has recently emerged as a prospect for improving yield of forage species. GBS can provide the high-density markers typically needed for GS based complex traits selection in forage species (Hayes et al., 2013; Baral et al., 2018). Thus, GS can enhance the rate of genetic gain by reducing the length of a breeding cycle and increasing selection accuracy (Fu et al., 2017).

GS can be performed with four major steps in a breeding population (Bassi et al., 2016). First, a training population and a test population need to be developed from a breeding population. Second, each individual genotypes in the training population will be both genotyped and phenotyped for traits of interest. A GS model will be applied to estimate genetic effects of marker data based on the trait measurements. Third, the individuals in the test population will be genotyped only. Using the estimated marker effects from the training population, the same GS model will be applied to estimate individual GEBVs in the test population. Fourth, selecting parental lines in the test population can be conducted using individual GEBV estimates. Several GS models have been proposed for the estimation of GEBV (Fu et al., 2017) such as best linear unbiased prediction (Henderson, 1975) and a Bayesian framework (Gianola and Fernando, 1986), and most of them are capable of capturing additive genetic variance for a trait of breeding interest. This is important as most of the target traits are genetically complex and involved with genes of small effects, for which traditional breeding approaches are less effective in improvement.

### Application of Genomic Selection in Bromegrass Breeding

GS would potentially be useful in improving forage yield of bromegrass. A single selection cycle in perennial forages requires multiple years of measurement for biomass yield, persistence, and quality traits, and at least 10–15 years are commonly required to release a new cultivar (Casler and Brummer, 2008). Yields and most other agronomically important traits in forages are quantitative and highly polygenic and require both labor and time intensive measurements (Wilkins and Humphreys, 2003). A number of other limitations that lead to a slow progress in bromegrass breeding include the use of recurrent selection in cultivar development as bromegrass is an obligate out-crossing grass, inability to exploit true heterosis, and generally a weak association between forage yield and quality (Casler and Brummer, 2008; Conaghan and Casler, 2011). Moreover, plant abiotic stress tolerance is also considered as a complex physiological mechanism controlled by a large number of genes (McDowell et al., 2008). Given such a complicated breeding target for bromegrass species with traditional breeding approaches, GS can be a feasible alternative to accelerate genetic gains in forage yield and other complex traits of bromegrasses. In particular, GS may be useful for improving bromegrass adaptation to climate change as most of the physiological traits have low heritability and GS is capable of capturing gene effects at both low heritable and small-effect quantitative trait loci. Another possible complication is associated with the presence of genotype × environment interaction in multi-location breeding trials that creates difficulty in the selection of stable lines across different growth environments. However, advanced GS models can effectively handle this complex situation without compromising selection accuracy. For instance, a number of researchers have used specific GS models with genomic main effects and genotype × environment interactions, including sets of environmental co-variables, to achieve an increased prediction accuracy (Burgueno et al., 2012; Jarquin et al., 2014). However, for the application of GS with an increased accuracy of GEBV in bromegrass breeding, a careful consideration should be given to a number of factors such as linkage disequilibrium, relationship between training and breeding populations, population size, number and types of markers, traits, and plant breeding schemes (Nakaya and Isobe, 2012).

The feasibility of GS as a breeding tool has been recently investigated with some encouraging findings (**Table 6**). One of them was done by Faville et al. (2018) to examine the rate of genetic gain for forage yield of perennial ryegrass using GBS in a multi-population training set of five populations phenotyped as half-sib families in five environments over 2 years. GBS was conducted using the *Ape*KI enzyme, which yielded 1.02 million single nucleotide polymorphism markers from a training set of *n* = 517 genotypes. The multi-population-based genomic prediction models for forage yield were made in all five populations and cross-validation predictive ability ranged from 0.07 to 0.43 by trait and target population (**Table 6**). Best linear unbiased predictor based prediction methods were marginally superior or equal to ridge regression and random forest computational approaches. These results showed that GS resulted in a two-fold increase in genetic gain for forage yield of perennial ryegrass in a single selection cycle when applying a prediction model with moderate predictive ability and by combining among and within half-sib family selection. In another study, the predictive potentials of GS based on GBS data for agronomic and quality traits in alfalfa (*Medicago sativa* L.) were assessed in a total of 322 genotypes from 75 alfalfa accessions using BayesA, BayesB, and BayesC_Π_ methods (Jia et al., 2018). The results indicated that the above three genomic prediction methods displayed similar prediction accuracies for each trait. Overall, the prediction accuracies of GS for agronomic traits were higher than that for quality traits. Among 15 quality traits, the mineral element, calcium showed the highest accuracy (0.34) followed by NDF digestibility after 48 and 30 h (0.20 to 0.25) and the lowest accuracy found for fat and crude protein (0.05 to 0.19). Among 10 agronomic traits, the prediction accuracies for plant height in fall, flowering date, and plant regrowth were 0.65, 0.52, and 0.51, respectively. The accuracies for leaf-to-stem ratio, plant branching, and biomass yield ranged from 0.25 to 0.32. These studies, together, suggest that GS is successful in alfalfa and perennial ryegrass breeding (**Table 6**). However, bromegrass (allo-auto-octoploid) is much more genetically complex compared to ryegrass (diploid) and alfalfa (auto-tetraploid) (Baral et al., 2018). High density genetic markers along with appropriate plant breeding schemes may be considered to address genetic complexity and to increase prediction accuracy of target traits in bromegrass.

**Table 6:** Published applications of genomic selection techniques to crop genetic improvement.

Traits predicted	Crop	Population	Size of training population	Markers	Statistical model	Prediction accuracy	Reference
Biomass, feed quality	Perennial ryegrass	HSP	364	1670	BLUP	0.10–0.59	Grinberg et al. (2016)
Grain yield, green leaf area	Bread wheat	FSF	613	9285	BLUP	0.56–0.62	Juliana et al. (2019)
Stem NDF digestibility, leaf protein content	Alfalfa	HSP	154	8,494	BLUP, BayesB, and Bayesian Lasso	0.3.0–.0.4.0	Biazzi et al. (2017)
Plant height, flowering date, plant regrowth	Alfalfa	G	288	44,757	BayesA, BayesB, and BayesC	0.51–0.65	Jia et al. (2018)
Days to heading (DH), herbage accumulation (HA)	Perennial ryegrass	HSP	517	1.02 ×10^5^	BLUP and GBLUP	0.40–0.52 (DH) 0.07–0.43 (HA)	Faville et al. (2018)

HSP, half-sib progeny; FSF, full-sib family; G, genotype.

## Perspectives

An increase in genetic gain in forage yields of bromegrass is required to meet the projected demand for forage yields in the future (Casler et al., 2000; Vogel and Hendrickson, 2018). Selection for improved photosynthetic related traits and radiation-use efficiency may increase forage yield of bromegrass. Also, an improvement of bromegrass adaptation to changing environments may increase forage availability and stability under future climate conditions. Availability of high-throughput imaging technologies and open-sourced data extraction and analysis tools have made it feasible to screen a large number of genotypes for complex physiological traits related to growth, yield, quality, resource-use efficiency, and abiotic stress tolerance in both field and laboratory conditions. With these advances in high-throughput phenotyping technologies, GS would be more effective in improving complex traits compared to conventional breeding methods. We reason that these advanced technologies hold promise in advancing bromegrass breeding and may accelerate the development of high-yielding and climate-resilient bromegrass cultivars.

## Ethics Statement

The writing process of the manuscript complies with current laws of Canada.

## Author Contributions

DB conducted the literature review and wrote the manuscript. BC revised the manuscript. BB conceived the research and revised the manuscript and Y-BF conceived the research and revised the manuscript.

## Funding

The work was financially supported by Beef Cattle Research Council of Canada (BCRC) granted to BB and Y-BF.

## Conflict of Interest

The authors declare that the research was conducted in the absence of any commercial or financial relationships that could be construed as a potential conflict of interest.
